# How aging anxiety relates to self-rated health in middle-aged and older adults: the role of psychological pathways

**DOI:** 10.3389/fpsyg.2026.1782428

**Published:** 2026-02-19

**Authors:** YuXin Zhou, DanDan Chen, Bo Dong, MengHan Jiang, YiFan Li

**Affiliations:** 1 School of Marxism, Xi'an Jiaotong University, Xi'an, China; 2 Business School, University of International Business and Economics, Beijing, China; 3 School of Public Health, Zhejiang Chinese Medical University, Hangzhou, China; 4 School of Humanities and Management, Zhejiang Chinese Medical University, Hangzhou, China; 5 Shandong Cancer Hospital and Institute, Shandong First Medical University and Shandong Academy of Medical Sciences, Jinan, China

**Keywords:** aging anxiety, buffering effect, health, heterogeneity, middle-aged and older adults

## Abstract

**Objective:**

Aging anxiety is not only a critical practical issue for understanding the psychology of middle-aged and older adults, but also a key pathway for improving their health levels, optimizing aging policies, and achieving active and healthy aging. Existing research has insufficiently addressed the pathways and heterogeneity through which aging anxiety impacts health. This study aims to reveal the effects of aging anxiety on the health of middle-aged and older adults, its underlying mechanisms, and the differences in its impact across various groups.

**Methods:**

Drawing on large-scale data from the 2021 China General Social Survey (CGSS), this study first employs a logistic regression model to analyze the impact of aging anxiety on the health of middle-aged and older adults. Then, we employ structural equation modeling to examine the mediating roles of psychological pessimism, sleep disturbance, and loss of self-efficacy in this relationship. Subsequently, a moderation analysis examines the buffering effects of six factors—including social participation and social support—on the relationship between aging anxiety and health outcomes. Finally, heterogeneity analysis explores variations in aging anxiety impacts across different groups defined by income, household registration status, education level, and age.

**Results:**

Higher aging anxiety is significantly associated with poorer self-rated health among middle-aged and older adults (*β* = −0.271). Mediation analyses suggest that this association may be partially mediated by psychological pessimism, sleep disturbance, and loss of self-efficacy. Psychological pessimism accounts for 26.53% of the total indirect effect, sleep disturbance for 8.21%, and loss of self-efficacy for 27.22%. Moderation analyses indicate that social participation, social support, social capital, healthcare utilization, insurance coverage, and digital technology use attenuate the negative association between aging anxiety and health. Heterogeneity analyses further show that the negative association is stronger among low-income, rural, less-educated, and older respondents.

**Conclusion:**

These findings highlight the potential value of a multidimensional coping framework to address aging-related concerns and support healthy aging. Such efforts may include fostering more positive perceptions of aging, strengthening psychologically informed and behaviorally oriented supports (e.g., pessimism reduction, sleep management, and self-efficacy enhancement), improving social and institutional resource support systems, and prioritizing targeted strategies for vulnerable and older populations.

## Introduction

As the population ages at an accelerating pace, the physical and mental health of middle-aged and older adults has become an increasingly urgent concern. Aging is not merely a natural biological process but also a psychological and social experience shaped by sociocultural contexts (Johfre and Saperstein, 2023; Gilleard, 2025). Amidst the interplay of traditional beliefs and modern discourse, “growing old” is often stigmatized with negative connotations such as functional decline, social marginalization, and diminished value. This has, to some extent, heightened the sensitivity and insecurity among middle-aged and older adults regarding their own aging process (Bergman and Segel-Karpas, 2021; Chasteen et al., 2021). Particularly against the backdrop of medical advances, extended average life expectancy, and significantly lengthened later years, people’s concerns about “how to age” and “whether to age healthily and with dignity” have gradually surpassed the singular pursuit of “how long to live.” The accompanying issue of aging anxiety has become increasingly prominent.

“Aging anxiety” refers to the persistent negative emotions and cognitive experiences—such as tension, unease, and worry—that individuals develop when they perceive potential adverse changes in their physiological functions, physical appearance, social roles, and interpersonal resources as they grow older (Sargent-Cox et al., 2014; Barnett and Adams, 2018). This anxiety stems both from concerns about declining physical function and increased chronic disease risks, as well as worries about insufficient economic security, reduced social roles, diminished family support, and age discrimination. Compared to other age groups, middle-aged and older adults are particularly susceptible to aging anxiety due to the distinctive characteristics of their life stage. On one hand, they commonly face health vulnerabilities such as multiple coexisting chronic conditions and gradual physical decline, where even minor physical discomforts can be amplified into fears of “irreversible aging” (García-García et al., 2025; Chowdhury et al., 2023). On the other hand, they shoulder multiple responsibilities—supporting elderly parents, raising children (or even grandchildren), and maintaining family and social relationships. With diminishing resources and waning energy, they are more prone to experiencing feelings of being “overwhelmed” (Chai et al., 2021). Additionally, retirement systems, shifts in generational structures, the digital divide, and reduced opportunities for social engagement collectively shape the unique life context of middle-aged and older adults. These specificities render aging anxiety in this demographic not only widespread but also imbued with distinct generational and group characteristics; its pathways of impact on health are likely more complex and multifaceted. Moreover, compared to general anxiety, aging anxiety—centered on the “age-aging” narrative—exhibits greater persistence and situational sensitivity. It is frequently triggered by subtle cues in daily life and is deeply intertwined with an individual’s self-concept and identity. Consequently, it subtly shapes the health perceptions and lifestyles of middle-aged and older adults (Wurm et al., 2017; Silva-Smith and Benton, 2021; Gao et al., 2024). However, these very characteristics of “normalization” and “covertness,” combined with the unique physical, psychological, and social roles of middle-aged and older adults, highlight the need for more systematic research to understand aging anxiety’s true impact on their health and its underlying mechanisms.

Regarding aging anxiety and health, while existing research has examined aging anxiety and its health consequences, suggesting that aging anxiety negatively impacts health (Yang and Ge, 2025; Luo et al., 2025), there remains room for improvement in current studies. First, existing research tends to focus on “describing outcomes” while neglecting “mechanism analysis.” Much of the existing research is largely correlational, linking aging anxiety to outcomes like depression and life satisfaction without providing sufficient mechanistic insight into how it affects the health of middle-aged and older adults. Clear models of the mediating or moderating pathways remain elusive. Second, existing studies often treat research subjects as a relatively homogeneous “whole group,” paying insufficient attention to internal population differences. Third, practical implications remain insufficiently explicit. Current studies often offer only broad recommendations (e.g., “enhance psychological counseling”), lacking concrete, evaluable strategies grounded in specific mechanistic evidence.

In recent years, China has been actively advancing strategies for “active aging” and “healthy aging.” Through policy measures such as the Healthy China Initiative, the development of integrated medical and elderly care systems, and the implementation of digital assistance services for seniors, the country emphasizes ensuring that the elderly “have support in old age, remain engaged, continue learning, and find joy.” This approach encourages middle-aged and older adults to actively embrace society, maintain vitality, and realize their value, providing new institutional support for their mental health and social integration. However, age discrimination remains widespread in practice. Support systems for the mental health and health education of middle-aged and older adults remain inadequate. The concepts of “active participation” and “proactive health management” in aging have not yet been fully embraced. This has, to some extent, limited the effective implementation of the active and healthy aging philosophy. Therefore, investigating how aging anxiety affects the health of middle-aged and older adults is essential for developing effective positive aging strategies and promoting psychological resilience and healthy behaviors in this population. This study employs large-scale cross-sectional survey data from China to investigate the effects of aging anxiety on the health of middle-aged and older adults and its underlying mechanisms, while also analyzing the heterogeneity of these effects across multiple dimensions. We anticipate that these analyses will not only provide evidence for alleviating aging anxiety among middle-aged and older adults but also offer support for achieving active and healthy aging.

## Methodology

### Data sources

The data utilized in this study are drawn from the 2021 Chinese General Social Survey (CGSS). The CGSS is a large-scale, continuous, random-sample survey project initiated in 2003 by the China Survey and Data Center at Renmin University of China (Cheng and Mao, 2024). The survey boasts a comprehensive sample covering 19 provinces, autonomous regions, and municipalities directly under the central government. It employs a multi-stage stratified sampling approach, with counties serving as primary sampling units. Post-stratification weights are applied to correct oversampling, ensuring that survey results accurately represent the general population in China (Yang et al., 2021).

The survey encompasses multiple levels—society, community, household, and individual—and covers diverse dimensions including economic, political, social, cultural, institutional, behavioral, and attitudinal aspects, providing rich data for studying economic conditions and individual mentalities. Most significantly, the 2021 survey includes a module on respondents’ aging anxiety, offering extensive information absent from other comparable surveys and providing robust data support for related research on aging anxiety. The 2021 survey collects a total of 8,148 valid samples nationwide. After handling missing values in the samples, 1,762 samples are retained for this study. The specific sample selection process is shown in Appendix Figure 1.

**Figure 1:**
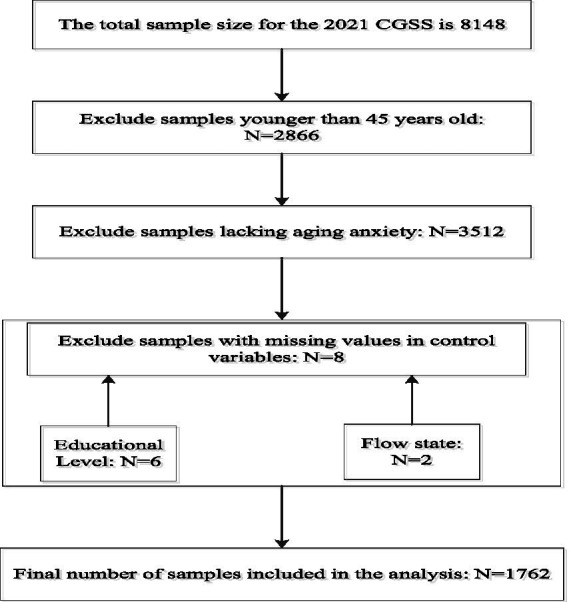
Sample inclusion exclusion flowchart.

It should be noted that all variables in this study are drawn from the CGSS 2021 questionnaire, which may raise concerns about common method variance (CMV). While this limitation is inherent to secondary survey data, our key constructs are measured with distinct item sets from different questionnaire sections, and our analyses include a broad set of covariates to mitigate this concern. Nevertheless, CMV cannot be fully ruled out, and the results should be interpreted as associations (Figure 1).

### Variables selection

#### Dependent variables

The dependent variable of interest in this study is health status, with self-assessed health employed to measure the health condition of the migrant population. Self-assessed health status has been reported to correlate with an individual’s actual health level (Wu et al., 2013; Wang and Cao, 2024), serving as a cross-contextually applicable proxy for actual health status. In the CGSS2021 questionnaire, the specific question regarding dependent variables is “How would you rate your current physical health status?” Responses comprise five possible outcomes: “Very unhealthy,” “Somewhat unhealthy,” “Average,” “Somewhat healthy,” and “Very healthy.” In this study, we group these five outcomes into three categories: “1 = Unhealthy,” encompassing “Very Unhealthy” and “Somewhat Unhealthy”; “2 = Average Health”; and “3 = Healthy,” covering “Somewhat Healthy” and “Very Healthy.” Lower self-rated health values indicate poorer health levels, while higher values signify better health status.

It can be seen that when using self-rated physical health from CGSS 2021 as the dependent variable in this study, the original item is a 5-level ordinal scale. To address potential small-sample issues in extreme categories and to ensure robust, interpretable estimates for mechanism and interaction analyses, the original categories are merged into three. This approach preserves the hierarchical information of health levels while reducing estimation instability risks caused by category sparsity, facilitating subsequent model comparisons and result presentation. In regression analysis, we estimate the association between aging anxiety and health levels. For mechanism testing, structural equation modeling is employed to depict the mediating structure of “aging anxiety → psychological pathways → health.” Additionally, we conduct robustness checks by re-estimating our models using the original 5-level self-rated health measure to verify the consistency of our conclusions, which strengthens the robustness and credibility of the findings.

#### Independent variables

The independent variable in this study is “aging anxiety.” Due to the absence of comprehensive measurement tools such as the Aging Anxiety Scale (AAS) or Personal Anxiety Toward Aging Scale (PAAS) in large-scale Chinese surveys, we employ a validated alternative approach based on three questions from the health module of the CGSS 2021 survey. These items reflect concerns about physical disability/mobility limitations, loss of cognitive ability or decision-making autonomy, and financial dependence—key themes also present in existing aging anxiety scales. Following established methodology, we construct a continuous variable by averaging responses to the following three items: “I worry that I won’t be able to take care of myself when I get older.” “I worry that I will have to let others make decisions for me when I get older.” “Financial dependence on others is one of my greatest concerns about aging.” For each statement, respondents are asked: “To what extent do you agree with this statement?” Responses and corresponding scores are as follows: Strongly disagree = 0, Disagree = 1, Undecided/Neither agree nor disagree = 2, Agree = 3, Strongly agree = 4. The continuous value for this variable is derived by calculating the average score across these three responses.

It should be noted that since CGSS 2021 does not include psychometrically validated standard scales for aging anxiety (such as the AAS or PAAS), this study adopt a method consistent with relevant research (Luo et al., 2025). We select three highly correlated self-report items from the questionnaire to construct a substitute indicator, reflecting concerns about functional decline, loss of autonomy, and economic dependence. This indicator primarily captures core concerns related to independence/capability within aging anxiety, demonstrating conceptual consistency with the key dimensions of established scales. Thus, it serves as a proxy variable for aging anxiety. However, its construct coverage remains limited, and related conclusions should be interpreted within the scope of this measurement.

#### Mediating variables

Drawing upon relevant research (Craig et al., 2023; Barnett and Anderson, 2020; Becker et al., 2018; Du et al., 2023) and data availability, we identify psychological pessimism, sleep disturbance, and loss of self-efficacy as mediating variables through which aging anxiety impacts the health of middle-aged and older adults. Together, these three factors constitute a typical psychosocial pathway: “subjective cognition—physiological function—behavior and resources” (see Appendix 1 for detailed measurement methods of the three mediating variables). On one hand, aging anxiety heightens negative expectations about aging, causing middle-aged and older adults to interpret their physical changes and social circumstances more pessimistically. This psychological pessimism not only directly undermines their subjective wellbeing and quality of life but also diminishes their confidence and motivation to maintain health. On the other hand, prolonged anxiety and pessimism disrupt sleep structure and quality through mechanisms like heightened arousal and excessive worry, leading to sleep disturbance. As a vital physiological process, sleep disturbance undermines recovery, worsens emotional and physical distress, and compounds the detrimental health effects of anxiety. In this process, loss of self-efficacy serves as a pivotal link: aging anxiety and its associated pessimistic experiences reduce middle-aged and older adults’ confidence in managing aging risks, disease control, and maintaining healthy behaviors. This reduces their engagement in proactive health behaviors—such as seeking medical care, exercising, and self-care—translating manageable health risks into actual health problems. Therefore, psychological pessimism, sleep disturbance, and loss of self-efficacy not only align theoretically with the classic stress-cognitive appraisal-physiological and behavioral response framework but have also been repeatedly confirmed in empirical studies as closely related to older adults’ health. Incorporating these factors into the analysis of mediating mechanisms in the aging anxiety-health relationship helps reveal in greater detail how aging anxiety transforms from a “psychological experience” into “health outcomes.” This provides a basis for designing precise psychological interventions and health promotion pathways.

#### Moderating variables

The impact of aging anxiety on the health of middle-aged and older adults may also be moderated by other factors. Identifying the mediating factors and assessing their buffering or amplifying effects can clarify the underlying heterogeneity in how aging anxiety impacts health. This provides a scientific basis for targeted interventions. To achieve these objectives, this study selects social participation, social support, social capital, healthcare utilization, insurance participation, and digital technology use as moderating factors, based on the relevant literature and data availability. (Zhang et al., 2020; Ai et al., 2024; Jiang and Jiang, 2023; Chang et al., 2023; Norbye et al., 2022; Yang et al., 2023; Yang et al., 2024) (see Appendix 1 for detailed measurement methods of the six moderating variables). Together, these factors form a “multi-level resource system” that spans micro to macro levels, relational networks to institutional safeguards, and offline life to digital spaces. On one hand, social participation and social support represent the degree to which middle-aged and older adults maintain ongoing social connections. This shapes whether individuals withdraw socially or reaffirm their worth through interaction and recognition when facing the threat of aging. Social capital, operating at the macro level of network and trust structures, influences an individual’s capacity to mobilize diverse resources and transform aging anxiety into motivation for proactive coping. Healthcare utilization and insurance participation, on the other hand, represent the strength of institutional safety nets, which dictates whether the health risks associated with aging anxiety can be detected, managed, and afforded. Digital technology adoption serves as a critical variable in the digital age, determining whether older adults can “access” new health information and service systems, bridge the digital divide, and avoid further marginalization in modern society. Therefore, these six factors are not merely parallel background variables. Together, they shape whether aging anxiety escalates into a health crisis or is channeled into an adaptive process supported by adequate resources and tools. This reveals that the essence of aging anxiety’s impact on middle-aged and older adults’ health is a “conditional effect” deeply constrained by resource allocation, institutional arrangements, and technological environments. This provides a clear basis for understanding health disparities and designing tiered interventions. For specific measurements of relevant mediating and moderating variables, please refer to Appendix 1.

#### Control variables

This study selects control variables based on Anderson’s Health Care Utilization Model (Seidu, 2020; Park, 2025) while considering data availability. These variables, used to adjust for confounding effects, are categorized into three types: propensity characteristics, enabling resources, and situational characteristics. Propensity characteristics include gender, age, marital status, religious affiliation, ethnicity, and educational attainment (Li and Dou, 2022); enabling resources encompass income level, household registration type, employment status, number of children, and housing conditions (Tian et al., 2022); and situational characteristics represent mobility patterns (Su et al., 2023). Given regional variations in China’s economic, social environments, and medical insurance policies—which may influence respondents’ health outcomes (Zhu et al., 2022)—this study also controls for residential region within propensity characteristics, following relevant literature (Wang et al., 2021). Definitions and values for each variable, based on the selected data conditions, are presented in Table 1.

**Table 1:** Variable definition.

Variables		Assignment
Dependent variable	Health status	Unhealthy = 1; Normal = 2; Health = 3
Independent variable	Aging anxiety	Continuous variable
Mediating variables	Psychological pessimism	Never = 0; Rarely = 1; Sometimes = 2; Often = 3; Always = 4
	Sleep disorder	Very good = 0; Relatively good = 1; Relatively poor = 2; Very poor = 3
	Loss of self-efficacy	Very confident = 0; Confident = 1; Neither confident nor unconfident = 2; Unconfident = 3; Very unconfident = 4
Moderating variables	Social participation	Continuous variable
	Social support	No = 0; Yes = 1
	Insurance participation	Participation = 1; Non-participation = 0
	Healthcare service utilization	Never = 0; About once a year = 1; Several times a year = 2; About once a month = 3; About once a week = 4; Several times a week = 5
	Digital technology usage	Never = 1; Rarely = 2; Sometimes = 3; Often = 4; Very frequently = 5
	Social capital	Strongly disagree = 1; Disagree = 2; Neither agree nor disagree = 3; Agree = 4; Strongly agree = 5
Control Variables	Age	45–60 = 1; ≥ 60 = 2
	Ethnic	Han ethnicity = 1; Ethnic minorities = 2
	Gender	Male = 1; Female = 2
	Religious belief	No = 0; Yes = 1
	Educational attainment	Junior high school and below = 1; High school = 2; College and above = 3
	Marital status	Unmarried = 1; Married = 2
	Household registration	Rural = 1; Urban = 2
	Number of children	≤3 = 1; 3–6 = 2; ≥ 6 = 3
	Living conditions	How serious are the following issues in the area where you live? This variable is a continuous variable by calculating the mean score of the responses to the following statements. “What is the severity of air pollution in your place of residence?” “What is the severity of water pollution in your place of residence?” “What is the severity of noise pollution in your place of residence?” “What is the severity of insufficient lighting in your place of residence?” For each question, the response options and their corresponding scores are: Not severe at all = 0; Somewhat severe = 1; Severe = 2; Very severe = 3. Take the average of the values for each dimension across the three questions.
	Income	≤50,000 = 1; 50,000–100,000 = 2; ≥100,000 = 3
	Region	Western Region = 1; Central Region = 2; Eastern Region = 3
	Work	No work = 1 Work = 2
	Flow state	Non-migrant population = 1; Migrant population = 2

### Statistical analysis

In this study, we employ Stata 22.0 software for data organization and analysis. For descriptive analysis, categorical variables are presented using frequencies and percentages to characterize participants, while continuous variables are described using means and standard deviations. We use chi-square tests to assess the statistical significance of health status differences across demographic subgroups. Subsequently, to examine the relationship between aging anxiety and health, a logistic regression model is utilized to evaluate the impact of aging anxiety on health outcomes.

Using structural equation modeling to explore the association between aging anxiety and respondent health, with psychological pessimism, sleep disturbance, and loss of self-efficacy serving as mediating variables. We apply a bootstrap method, calculating bias-corrected (BC) 95% confidence intervals (CIs). Total, direct, and indirect effects are estimated by resampling 2000 times from the initial sample in the structural equation model analysis. Effects are deemed statistically significant (*p* ≤ 0.05) if their 95% confidence intervals exclude zero.

**Figure 2:**
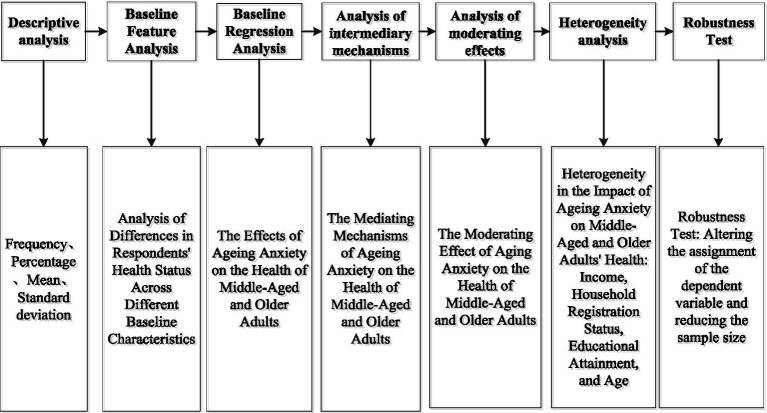
The flow chart of the study.

The moderation effect model is employed to examine the moderating roles of “social participation, social support, social capital, healthcare utilization, health insurance coverage, and digital technology” between aging anxiety and health status. Finally, in the robustness testing section, to assess the reliability of the findings, this study conducts robustness analyses by altering the assignment of the dependent variable and reducing the sample size. The research process is illustrated in Figure 2.

### Research findings

#### Characteristics of respondents

Table 2 presents descriptive statistics for key variables in this study. Among the 1,762 respondents, males and females comprise for 46.2 and 53.8%, respectively. Middle-aged adults (45–60 years) comprise 44.0% of the sample, while older adults (≥60 years) make up the remaining 56.0%. Educational attainment is generally low: only 18.10% hold a college degree or higher, while 71.90% have a high school education or below. Rural household registration is held by 60.10% of the population, with only 39.90% originating from urban areas. Regarding religious beliefs, 90.40% of respondents do not profess any religious faith. Analyzing personal annual income, 15% of respondents earn over 100,000 yuan annually. Regional distribution shows 55.73% of respondents come from eastern regions, while those from central and western regions account for 27.64 and 16.63%, respectively. The mean for respondents’ living conditions is 0.86, with a standard deviation of 0.75.

**Table 2:** Analysis of basic sample characteristics.

Variables		*N*	%
Age	45–60	775	44.00%
	≥60	987	56.00%
Ethnic	Han ethnicity	1,635	92.80%
	Ethnic minorities	127	7.20%
Gender	Male	814	46.20%
	Female	948	53.80%
Religious belief	No	1,592	90.40%
	Yes	170	9.60%
Educational attainment	Junior high school and below	937	53.20%
	High school	506	28.70%
	College and above	319	18.10%
Marital status	Unmarried	453	25.70%
	Married	1,309	74.30%
Household registration	Rural	1,059	60.10%
	Urban	703	39.90%
Number of children	≤3	1745	99.00%
	3–6	13	0.70%
	≥6	4	0.20%
Income	≤50,000	1,058	60.00%
	50,000–100,000	439	24.90%
	≥100,000	265	15.00%
Region	Western region	293	16.63%
	Central region	487	27.64%
	Eastern region	982	55.73%
Work	No work	993	56.40%
	Work	769	43.60%
Flow state	Non-migrant population	1,342	76.20%
	Migrant population	420	23.80%
Living conditions		0.86	0.75

#### Analysis of health status differences among participants in the baseline survey

Univariate analysis is conducted using respondents’ health status as the dependent variable, with results presented in Table 3. Statistically significant differences in health levels are observed across various demographic factors, including age, education level, marital status, household registration, income, region, occupation, migration status, and living conditions.

**Table 3:** Analysis of differences in respondents’ health status across various characteristics.

Variables		Unhealthy		Normal		Health		Chi-square	*p*
		*N*	%	*N*	%	*N*	%		
Age	45–60	95	12.3	207	26.7	473	61	25.494	0.000
	≥60	184	18.6	314	31.8	489	49.5		
Gender	Male	129	15.8	235	28.9	450	55.3	0.38	0.827
	Female	150	15.8	286	30.2	512	54		
Ethnic	Han ethnicity	259	15.8	259	15.8	889	54.4	0.571	0.752
	Ethnic minorities	20	15.7	34	26.8	73	57.5		
Ethnic	No	253	15.9	469	29.5	870	54.6	0.109	0.947
	Yes	26	15.3	52	30.6	92	54.1		
Educational attainment	Junior high school and below	202	21.6	393	41.9	342	36.5	293.027	0.000
	High school	36	0.071	53	0.105	417	0.824		
	College and above	41	0.129	75	0.235	203	0.636		
Marital Status	Unmarried	92	0.203	134	0.296	227	0.501	9.959	0.007
	Married	187	0.143	387	0.296	735	0.561		
Household registration	Rural	220	20.8	335	31.6	504	47.6	68.591	0.000
	Urban	59	8.4	186	26.5	458	65.1		
Number of children	≤3	278	15.9	516	29.6	951	54.5	4.234	0.375
	3–6	0	0	5	38.5	8	61.5		
	≥6	1	25	0	0	3	75		
Income	≤50,000	231	21.8	451	42.6	376	35.5	392.26	0.000
	50,000–100,000	34	7.7	51	11.6	354	80.6		
	≥100,000	14	5.3	19	7.2	232	87.5		
Region	Western region	24	0.1	90	0.377	125	0.523	18.157	0.001
	Central region	69	0.142	130	0.267	288	0.591		
	Eastern region	186	0.18	301	0.291	549	0.53		
Work	No work	178	0.179	283	0.285	532	0.536	7.599	0.022
	Work	101	0.131	238	0.309	430	0.559		
Flow state	Non-migrant population	198	14.8	400	29.8	744	55.4	4.988	0.083
	Migrant population	81	19.3	121	28.8	218	51.9		
Living conditions		0.76	0.63	0.85	0.65	0.9	0.64	5.062	0.006

#### Analysis of the impact of aging anxiety on health

Logistic regression analyses are conducted with respondents’ health status as the dependent variable and aging anxiety as the key independent variable, adjusting for all covariates that are statistically significant in the univariate analyses. The results are reported in Table 4. Aging anxiety is significantly associated with poorer health status among middle-aged and older adults (*p* < 0.01). Overall, higher levels of aging anxiety were linked to a lower likelihood of reporting better health categories.

**Table 4:** Main regression analysis of the impact of aging anxiety on health.

Variables	
Aging anxiety	−0.271^***^
	(0.025)
≥60 (Reference group: 45–60)	0.647^***^
	(0.078)
High school (Reference group: Junior high school and below)	0.131^**^
	(0.064)
College degree or higher (Reference group: Junior high school or below)	0.303^***^
	(0.047)
Married (Reference group: unmarried)	0.111^***^
	(0.035)
Urban (Reference group: rural)	0.837^***^
	(0.079)
Income between 50,000 and 100,000 (Reference group: ≤50,000)	0.055
	(0.076)
Income over 100,000 (Reference group: ≤50,000)	−0.100
	(0.084)
Central region (Reference group: western region)	0.113^**^
	(0.047)
Eastern region (Reference group: western region)	0.226^***^
	(0.042)
Employment (Reference group: no employment)	0.078^**^
	(0.031)
Migrant population (reference group: non-migrant population)	−0.037
	(0.035)
Living conditions	−0.023
	(0.025)
Constant	2.372^***^
	(0.115)
Observations	1762
*R* ^2^	0.303
Adjusted *R*^2^	0.296

Standard errors in parentheses; ^*^p < 0.1, ^**^p < 0.05, ^***^p < 0.01.

#### Model fit indices

This study employs respondents’ aging anxiety as the independent variable, psychological pessimism, sleep disturbance, and loss of self-efficacy as mediating variables, and health status as the dependent variable. A basic mediational model with single-step multiple mediation is specified and tested for goodness of fit using AMOS 25.0. The basic model is revised based on the fit results. Table 5 presents the model fit indices. The model achieves a goodness-of-fit index (CFI) of 4.250 with a corresponding *p*-value greater than 0.05, indicating that the revised structural equation model fits the sample data well. Model fit indices are as follows: RMSEA = 0.015, CFI > 0.90, GFI > 0.90, and AGFI > 0.90. All indices fall within acceptable ranges, indicating a good overall fit between the data and the model. Consequently, this model can be used for path estimation.

**Table 5:** Model fitting indices.

Evaluation indicators	Model results	Compliance standards	Compatibility check
Chi-square value/degrees of freedom	1.417	<3.00	Yes
Chi-square value probability value *P*	0.236	>0.05	Yes
RMSEA	0.015	<0.08	Yes
CFI	0.996	>0.90	Yes
GFI	0.995	>0.90	Yes
AGFI	0.995	>0.90	Yes
NFI	0.997	>0.90	Yes

#### The logical structure of aging anxiety and health among middle-aged and older adults

**Figure 3:**
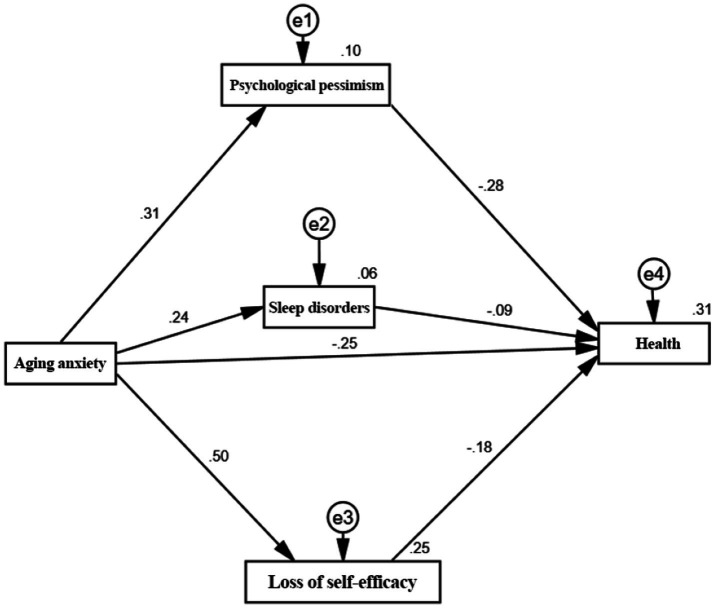
Mediating pathways through which aging anxiety affects health.

Next, are the coefficient estimates for the mediating paths. We employ the Bootstrap method with 5,000 repeated random samples and 95% confidence intervals, using the standard regression coefficients as the criterion to obtain estimates for the unidirectional paths. Since AMOS 25 does not display the significance levels for standardized results, the non-standardized significance levels are used to indicate overall significance. Figure 3 illustrates the causal pathways through which aging anxiety influences health, while Table 6 details the regression results with psychological pessimism, sleep disturbance, and loss of self-efficacy as mediating factors. The results indicate that aging anxiety exerts a significant positive direct effect on psychological pessimism (coefficient: 0.31), a significant positive effect on sleep disturbance at the 1% significance level (coefficient: 0.24), and a significant positive effect on loss of self-efficacy at the 1% significance level (coefficient: 0.50). Conversely, psychological pessimism, sleep disturbance, and loss of self-efficacy exerted significant negative effects on health status at the 1% significance level, with coefficients of −0.28, −0.09, and −0.18, respectively. This further indicates that aging anxiety exerts indirect effects on health through psychological pessimism, sleep disturbance, and loss of self-efficacy.

**Table 6:** Test results for mediating pathways of aging anxiety on middle-aged and older adults’ health.

Path	Non-standardization coefficient	Standardized coefficient	SE	CR	*P*
Aging anxiety → Health status	−0.159	−0.246	0.016	−10.136	***
Aging anxiety → Psychological pessimism	0.336	0.31	0.025	13.682	***
Aging anxiety → Sleep disorders	0.197	0.238	0.019	10.282	***
Aging anxiety → Loss of self-efficacy	0.527	0.502	0.022	24.362	***
Psychological pessimism → Health status	−0.168	−0.282	0.012	−13.541	***
Sleep disorders → Health status	−0.073	−0.094	0.016	−4.609	***
Loss of self-efficacy → Health status	−0.113	−0.184	0.014	−8.027	***

*** indicates significant at the 1% level of significance; ** indicates significant at the 5% level of significance; * indicates significant at the 10% level of significance.

### Testing the mediating effects of aging anxiety on health outcomes

To analyze the mechanism by which aging anxiety impacts health, this study further examines its specific pathways. The results of this pathway decomposition are presented in Table 7. It can be observed that in the relationship between aging anxiety and health, the indirect effect of psychological pessimism accounts for 26.53% of the total effect, while the indirect effect of sleep disturbance accounts for 8.21%. The indirect effect of loss of self-efficacy accounts for 27.22% of the total effect. Furthermore, the 95% confidence intervals (CIs) for the total, direct, and indirect effects exclude zero across all three mediators, thus confirming statistically significant mediation. This confirms that psychological pessimism, sleep disturbance, and loss of self-efficacy mediate the relationship between aging anxiety and health.

**Table 7:** Results of mediating effect tests.

Items	*c*	*a*	*b*	*a* * *b*	*a* * *b*		*c*′	Type of intermediary	Effect size percentage
	Overall effect			Mediation effect	(95% BOOT)		Direct effect		
					L	U			
Aging anxiety → Psychological pessimism → Health status	−0.334^***^	0.31^***^	−0.282^***^	−0.088^***^	−0.106	−0.074	−0.246^***^	Some intermediaries	26.35%
Aging anxiety → Sleep disorders → Health status	−0.268^***^	0.238^***^	−0.094^***^	−0.022^***^	−0.033	−0.015	−0.246^***^	Some intermediaries	8.21%
Aging anxiety → Loss of self-efficacy → Health status	−0.338^***^	0.502^***^	−0.184^***^	−0.092^***^	−0.117	−0.066	−0.246^***^	Some intermediaries	27.22%

*** indicates significant at the 1% level of significance; ** indicates significant at the 5% level of significance; * indicates significant at the 10% level of significance.

### Moderation effect analysis

To examine whether contextual resources condition the aging anxiety–health association, we estimate moderation models that include interaction terms between aging anxiety and six moderators: social participation, social support, social capital, healthcare utilization, insurance coverage, and digital technology use (Table 8). Aging anxiety is negatively associated with each moderator. Importantly, the interaction terms are positive and statistically significant, suggesting that higher levels of these resources weaken the negative link between aging anxiety and health. The negative link between aging anxiety and health is notably weaker among those reporting higher levels of social participation, support, capital, healthcare use, insurance coverage, and digital engagement. Overall, these findings are consistent with a buffering role of social and institutional resources in shaping vulnerability to aging-related concerns.

**Table 8:** Moderating effect analysis.

Variables	Social participation	Social support	Social capital	Medical services	Insurance enrollment	Digital technology
Aging anxiety	−0.290^***^	−0.283^***^	−0.301^***^	−0.309^***^	−0.345^***^	−0.319^***^
	(0.027)	(0.017)	(0.029)	(0.030)	(0.035)	(0.032)
Aging anxiety * Social participation	0.061^**^ (0.016)					
Aging anxiety * Social support		0.073^**^ (0.016)				
Aging anxiety * Social capital			0.021^**^ (0.009)			
Aging anxiety * Medical services				0.020^**^ (0.008)		
Aging anxiety * Insurance enrollment					0.095^***^ (0.032)	
Aging anxiety * Digital technology						0.035** (0.014)
≥60 (reference group: 45–60)	0.651^***^	0.632^***^	0.646^***^	0.624^***^	0.622^***^	0.650^***^
	(0.077)	(0.057)	(0.076)	(0.077)	(0.077)	(0.077)
High school (reference group: junior high school and below)	0.124^*^	0.134^*^	0.125^**^	0.095	0.113^*^	0.094
	(0.063)	(0.043)	(0.063)	(0.066)	(0.064)	(0.066)
College degree or higher (reference group: junior high school or below)	0.277^***^	0.247^***^	0.273^***^	0.137^**^	0.259^***^	0.211^***^
	(0.047)	(0.037)	(0.047)	(0.053)	(0.048)	(0.052)
Married (reference group: unmarried)	0.122^***^	0.122^***^	0.123^***^	0.102^***^	0.102^***^	0.108^***^
	(0.034)	(0.034)	(0.034)	(0.035)	(0.035)	(0.035)
Urban (reference group: rural)	0.830^***^	0.823^***^	0.824^***^	0.816^***^	0.810^***^	0.841^***^
	(0.078)	(0.048)	(0.077)	(0.078)	(0.078)	(0.078)
Income between 50,000 and 100,000 (reference group: ≤50,000)	0.065	0.061	0.061	0.093	0.074	0.094
	(0.075)	(0.071)	(0.075)	(0.077)	(0.075)	(0.077)
Income over 100,000 (reference group: ≤50,000)	−0.081	−0.072	−0.086	−0.065	−0.084	−0.066
	(0.084)	(0.064)	(0.084)	(0.085)	(0.084)	(0.085)
Central region (reference group: western region)	0.107^**^	0.117^**^	0.104^**^	0.114^**^	0.116^**^	0.119^**^
	(0.046)	(0.036)	(0.046)	(0.046)	(0.046)	(0.047)
Eastern region (reference group: western region)	0.217^***^	0.237^***^	0.211^***^	0.228^***^	0.230^***^	0.233^***^
	(0.041)	(0.031)	(0.041)	(0.041)	(0.041)	(0.042)
Employment (reference group: no employment)	0.079^***^	0.089^***^	0.080^***^	0.079^***^	0.074^**^	0.079^**^
	(0.031)	(0.021)	(0.030)	(0.031)	(0.031)	(0.031)
Migrant population (reference group: non-migrant population)	−0.040	−0.024	−0.041	−0.036	−0.035	−0.038
	(0.035)	(0.024)	(0.035)	(0.035)	(0.035)	(0.035)
Living conditions	−0.027	−0.017	−0.024	−0.025	−0.025	−0.022
	(0.025)	(0.015)	(0.025)	(0.025)	(0.025)	(0.025)
Constant	2.308^***^	2.368^***^	2.270^***^	2.349^***^	2.421^***^	2.389^***^
	(0.116)	(0.126)	(0.119)	(0.122)	(0.127)	(0.126)
Observations	1762	1762	1762	1762	1762	1762
*R* ^2^	0.320	0.353	0.325	0.320	0.315	0.311
Adjusted *R*^2^	0.313	0.343	0.317	0.312	0.307	0.303

Standard errors in parentheses.

^*^p < 0.1, ^**^p < 0.05, ^***^p < 0.01.

### Heterogeneity analysis

To examine heterogeneity in the aging anxiety–health association, we conduct subgroup analyses across four dimensions: income, household registration (hukou), educational attainment, and age. The results are presented in Table 9. The negative association between aging anxiety and self-rated health is stronger among low-income respondents than among high-income respondents. Similarly, this negative association is more pronounced among rural residents than among urban residents. By education, the negative association is stronger among respondents with a high school education or below than among those with a college education or higher. The negative association also varies by age: it is stronger for adults aged ≥60 years (*β* = −0.296, *p* < 0.01) than for those aged 45–60 years (*β* = −0.215, *p* < 0.01), indicating a more pronounced effect among older adults.

**Table 9:** Results of heterogeneity analysis.

Variables	Income			Household registration		Education		Age	
	Low-income	Middle-income	High-income	Rural	Urban	Low education	Higher education	45–50	≥60
Aging anxiety	−0.302^***^	−0.233^***^	−0.113^**^	−0.290^***^	−0.228^***^	−0.285^***^	−0.172^**^	−0.215^***^	−0.296^***^
	(0.038)	(0.022)	(0.012)	(0.033)	(0.039)	(0.027)	(0.067)	(0.039)	(0.033)
≥60 (reference group: 45–60)	0.505^***^	0.792^***^	0.754^***^	0.652^***^	0.252^***^	0.829^***^	0.201		
	(0.109)	(0.123)	(0.206)	(0.082)	(0.022)	(0.090)	(0.160)		
High school (reference group: junior high school and below)	−0.897	−0.092	0.026	0.143	0.139	−0.008	0.031	0.143	0.144
	(0.682)	(0.073)	(0.070)	(0.088)	(0.093)	(0.040)	(0.094)	(0.091)	(0.090)
College degree or higher (reference group: junior high school or below)	0.350^***^	0.250^***^	0.178^*^	0.338^***^	0.265^***^			0.310^***^	0.304^***^
	(0.058)	(0.048)	(0.097)	(0.065)	(0.068)			(0.067)	(0.066)
Married (reference group: unmarried)	0.105^**^	0.102^*^	0.165^**^	0.096^**^	0.145^***^	0.062	0.333^***^	0.172^***^	0.073
	(0.049)	(0.061)	(0.071)	(0.046)	(0.053)	(0.038)	(0.086)	(0.051)	(0.047)
Urban (reference group: rural)	0.785^***^	0.879^***^	0.751^***^			1.021^***^	0.403^**^	0.823^***^	0.623^***^
	(0.111)	(0.125)	(0.206)			(0.092)	(0.162)	(0.077)	(0.067)
Central region (reference group: western region)	0.133^**^	0.029	0.063	0.183^***^	0.025	0.115^**^	0.164	0.020	0.193^***^
	(0.064)	(0.085)	(0.098)	(0.064)	(0.067)	(0.050)	(0.131)	(0.068)	(0.064)
Eastern region (reference group: western region)	0.258^***^	0.188^**^	0.149^*^	0.264^***^	0.183^***^	0.217^***^	0.323^***^	0.196^***^	0.258^***^
	(0.058)	(0.077)	(0.084)	(0.057)	(0.060)	(0.045)	(0.109)	(0.061)	(0.057)
Employment (reference group: no employment)	−0.096^**^	−0.003	−0.091	−0.089^**^	−0.063	−0.051	−0.171^**^	−0.072	−0.085^**^
	(0.043)	(0.055)	(0.064)	(0.042)	(0.045)	(0.034)	(0.078)	(0.044)	(0.043)
Migrant population (reference group: non-migrant population)	−0.057	0.022	0.013	−0.064	0.010	−0.043	−0.080	0.016	−0.070
	(0.048)	(0.064)	(0.081)	(0.048)	(0.052)	(0.038)	(0.099)	(0.052)	(0.048)
Living conditions	−0.018	−0.091^**^	0.031	−0.016	−0.022	−0.013	−0.056	−0.025	−0.014
	(0.034)	(0.046)	(0.054)	(0.034)	(0.038)	(0.028)	(0.063)	(0.037)	(0.035)
Constant	2.610^***^	2.173^***^	2.005^***^	2.425^***^	3.091^***^	2.226^***^	2.865^***^	2.218^***^	3.106^***^
	(0.151)	(0.194)	(0.235)	(0.140)	(0.137)	(0.129)	(0.266)	(0.149)	(0.122)
Observations	1,058	439	265	1,059	703	1,443	319	775	987
*R* ^2^	0.199	0.160	0.126	0.315	0.215	0.322	0.195	0.300	0.306
Adjusted *R*^2^	0.186	0.134	0.070	0.304	0.197	0.314	0.155	0.284	0.295

Standard errors in parentheses; ^*^p < 0.1, ^**^p < 0.05, ^***^p < 0.01; Income heterogeneity: ≤50,000 defined as low income; 50,000–100,000 as middle income; ≥100,000 as high income. Educational attainment heterogeneity: High school or below as low educational attainment; college or above as high educational attainment.

### Robustness test

To assess the robustness of the main findings, we conduct two sensitivity analyses: (1) alternative coding of the dependent variable and (2) a restricted-sample analysis. For alternative coding, we retain the original five-category self-rated health measure and recode it from 1 (“Very unhealthy”) to 5 (“Very healthy”), then re-estimate the regression models using this outcome. The results are reported in Column (2) of Table 10. For the restricted-sample analysis, we exclude respondents aged 45–59 and re-estimate the models among adults aged 60 years and above; the results are shown in Column (3) of Table 10. In both robustness checks, the association between aging anxiety and health remains negative and statistically significant, demonstrating the robustness of the main conclusions to alternative coding and sample composition.

**Table 10:** Robustness tests.

Variables	(1)	(2)	(3)
	Baseline model	Change dependent variable	Reduce the sample size
Aging anxiety	−0.271^***^	−0.396^***^	−0.296^***^
	(0.025)	(0.038)	(0.033)
≥60 (reference group: 45–60)	−0.647^***^	−0.937^***^	
	(0.078)	(0.119)	
High school (reference group: junior high school and below)	0.131^**^	0.234^**^	0.144
	(0.064)	(0.098)	(0.090)
College degree or higher (reference group: junior high school or below)	0.303^***^	0.419^***^	0.304^***^
	(0.047)	(0.072)	(0.066)
Married (reference group: unmarried)	0.111^***^	0.142^***^	0.073
	(0.035)	(0.053)	(0.047)
Urban (reference group: rural)	0.837^***^	1.235^***^	
	(0.079)	(0.120)	
Income between 50,000 and 100,000 (reference group: ≤50,000)	0.055	0.041	0.088
	(0.076)	(0.116)	(0.107)
Income over 100,000 (reference group: ≤50,000)	0.100	0.215^*^	0.030
	(0.084)	(0.129)	(0.116)
Central region (reference group: western region)	0.113^**^	0.168^**^	0.193^***^
	(0.047)	(0.072)	(0.064)
Eastern region (reference group: western region)	0.226^***^	0.328^***^	0.258^***^
	(0.042)	(0.064)	(0.057)
Employment (reference group: no employment)	0.078^**^	0.102^**^	0.085^**^
	(0.031)	(0.047)	(0.043)
Migrant population (reference group: non-migrant population)	−0.037	−0.077	−0.070
	(0.035)	(0.054)	(0.048)
Living conditions	−0.023	−0.027	−0.014
	(0.025)	(0.039)	(0.035)
Constant	2.372^***^	3.550^***^	3.106^***^
	(0.115)	(0.175)	(0.122)
Observations	1,762	1,762	987
*R* ^2^	0.303	0.269	0.306
Adjusted *R*^2^	0.296	0.262	0.295

Standard errors in parentheses.

^*^p < 0.1, ^**^p < 0.05, ^***^p < 0.01.

## Discussion

This study examines the relationship between aging anxiety and self-rated health among middle-aged and older adults in China and explores potential psychological pathways and contextual conditions underlying this relationship. Using large-scale cross-sectional data from the CGSS 2021, our analysis suggests that higher aging anxiety is significantly associated with poorer self-rated health. This pattern aligns with prior evidence that aging anxiety is associated with reduced engagement in health-promoting behaviors and lower overall wellbeing. (e.g., Lee et al. (Lee and Sung, 2017)), and with China-based research highlighting aging anxiety as a practical concern in the context of healthy aging (Yang and Ge, 2025). Taken together, these findings indicate that efforts to improve later-life health must consider both traditional socioeconomic determinants and individuals’ own perceptions and concerns about aging. Importantly, given the cross-sectional design, the results should be interpreted as associations rather than causal effects, and reverse causality or unobserved confounding cannot be ruled out.

At the same time, the interpretation of our results should consider a measurement constraint. Due to limitations of the CGSS questionnaire, we construct a proxy measure of aging anxiety using three items capturing concerns most directly related to health within the aging anxiety construct (functional decline, loss of autonomy, and financial dependence). This proxy cannot fully replace multidimensional, psychometrically validated instruments such as the AAS/PAAS. In particular, it does not cover other domains often included in standard scales (e.g., appearance-related aging concerns, shifts in social relationships/status, or broader existential worries). Accordingly, the present findings more precisely reflect the association between “independence-risk-centered aging anxiety” and self-rated health, as well as related psychological pathways, rather than a complete characterization of all dimensions of aging anxiety. This limitation has two implications. First, if unmeasured domains of aging anxiety are also related to health, our estimates may be conservative. Second, the observed relationships may be domain-specific, driven primarily by concerns about independence and autonomy. Future research could further validate this proxy against standard measures (e.g., convergent/discriminant validity, dimensional structure, and measurement consistency across groups) and incorporate longitudinal or multi-source data to improve construct coverage and strengthen inference.

Regarding potential pathways, our results suggest that psychological pessimism, sleep disturbance, and loss of self-efficacy may help explain how aging anxiety is linked to poorer self-rated health. Compared with prior studies (Yang and Ge, 2025; Luo et al., 2025), our findings elucidate the psychological mechanisms that may underlie this association. Specifically, aging anxiety may orient individuals toward negative aging-related cues (e.g., functional decline and role weakening), fostering a stable pessimistic expectation framework. Such pessimistic cognitions may reduce positive evaluations of health behaviors, increase feelings of helplessness, and amplify threat-related interpretations of bodily signals, thereby increasing worry and somatic complaints (Wurm et al., 2013; Bergman and Segel-Karpas, 2021). In parallel, aging anxiety often involves rumination, hypervigilance, and excessive body monitoring—states that become particularly pronounced at night, leading to difficulty falling asleep, frequent awakenings, and non-restorative sleep. Poor sleep, in turn, is linked to impaired immune, metabolic, and emotion regulation processes and may exacerbate anxiety and depressive symptoms, creating a reinforcing cycle of distress and compromised health (Palagini et al., 2017; Riemann et al., 2023; Palmer and Alfano, 2017). Additionally, aging anxiety may be associated with loss of self-efficacy through expectations of declining capability and uncontrollable future outcomes, which could reduce persistence in healthy behaviors (e.g., exercise, diet management, and disease control) and constrain engagement in social activities and health-promoting resources (Grembowski et al., 1993; Sallis et al., 1988; Wurm et al., 2013). Overall, these results converge to indicate that psychological pessimism, sleep disturbance, and loss of self-efficacy are key pathways linking aging anxiety to poorer health.

We further examine moderating mechanisms to identify conditions that may buffer this association. The results indicate that social participation, social support, social capital, healthcare utilization, insurance coverage, and digital technology use attenuate the negative association between aging anxiety and self-rated health. Together, these resources may form a resilience system that operates across interpersonal, community, institutional, and technological levels. These moderating patterns indicate that the link between aging anxiety and health is contingent on available resources—it is attenuated where individuals have stronger social, institutional, and personal tools for support and self-management. Social participation may strengthen social ties and resource access; social support may enhance perceived security and coping capacity; institutional safeguards and healthcare access may reduce uncertainty and perceived risk; and digital inclusion may broaden channels for health information and social connection (Ma et al., 2020; Meng et al., 2025; Cohen and Wills, 1985; Zhao et al., 2022; Lin et al., 2025; Zhou et al., 2024). These findings provide practical clues for interventions that aim to reduce vulnerability by improving supportive environments and resource availability.

Finally, heterogeneity analyses indicate that the negative association between aging anxiety and health is more pronounced among low-income, rural, less-educated, and older groups. This pattern underscores that the health impacts of aging anxiety are not evenly distributed but are disproportionately concentrated among those with fewer resources and greater social disadvantage. These results provide actionable directions for geriatric health promotion: under limited resources, priority may be given to socioeconomically disadvantaged groups to reduce health inequality risks associated with aging anxiety.

### Policy recommendations

Based on the findings above, we propose the following policy implications: First, promote positive perceptions of aging and the continuity of social roles. It is crucial to actively promote the diversity and positivity of aging through media advocacy, age-friendly campaigns, and community lifelong learning programs, thereby correcting the misconception that aging equates to being a “burden” or “decline.” These initiatives enhance societal respect and acceptance of middle-aged and older adults, reducing the social roots of aging anxiety. Flexible retirement policies and age-appropriate employment programs can also be established to provide opportunities for older adults to continue participating in the labor market and social activities. These measures help maintain their social roles, thereby alleviating anxiety about functional decline during aging. Simultaneously, encouraging older adults to actively engage in community governance increases their sense of social participation and self-worth, further reducing age-related anxiety. Second, prioritize addressing key mediating mechanisms of aging anxiety. Since aging anxiety impacts health through pathways like psychological pessimism, sleep disturbance, and loss of self-efficacy, policies should implement targeted interventions addressing these mechanisms. First, routine psychological screenings and counseling services can be integrated into primary healthcare settings. Group-based cognitive behavioral training and emotional management courses should be implemented to help older adults manage negative emotions and reduce aging anxiety. Second, integrate sleep assessments into family doctor contract services, offering sleep hygiene guidance, schedule management, and necessary specialist referrals. This addresses prevalent sleep disturbance among middle-aged and older adults, thereby mitigating anxiety’s negative effects. Finally, enhance self-efficacy through tiered health education and actionable exercise/nutrition plans. For instance, implement point-based incentive programs for health behavior improvement, helping older adults regain a sense of control over the aging process through practical actions. Third, strengthen social resource support and mitigation mechanisms. Social support and resource safeguards can significantly alleviate the negative impacts of aging anxiety. Policies should build upon this foundation to enhance social support systems, thereby amplifying the effectiveness of alleviating aging anxiety. Accessibility and effectiveness of social support networks can be improved by enriching community interest groups, volunteer activities, and similar initiatives. Simultaneously, establish neighborhood mutual aid stations to foster mutual support among community members, strengthen social connections, and reduce feelings of social isolation. It is also crucial to enhance the accessibility and user-friendliness of healthcare services, particularly at the primary care level, to ensure equitable and convenient access for middle-aged and older adults. Optimize medical insurance reimbursement processes and enhance support for long-term care insurance. Finally, implement differentiated intervention measures for vulnerable groups. This study reveals that low-income groups, rural residents, and those with lower educational attainment exhibit heightened sensitivity to aging anxiety. Consequently, policies should prioritize interventions for these populations. Priority efforts should target low-income and rural older adults by enhancing the allocation of mental health services, digital infrastructure, and health education to improve their health literacy and capacity to cope with aging anxiety.

## Conclusion

This study uses data from the 2021 China General Social Survey (CGSS) to examine the association between aging anxiety and self-rated health among middle-aged and older adults. It further explores potential mediating and moderating processes, as well as heterogeneity across population groups. The main findings are as follows. First, higher aging anxiety is significantly associated with poorer self-rated health among middle-aged and older adults. Second, psychological pessimism, sleep disturbance, and loss of self-efficacy emerged as plausible mediating pathways that may help explain this association. Meanwhile, social participation, social support, social capital, healthcare utilization, insurance coverage, and digital technology use appeared to buffer the negative association between aging anxiety and health. Finally, the association exhibits significant heterogeneity, being most pronounced among socioeconomically disadvantaged and older adults, highlighting the need for targeted and prioritized interventions. Given the cross-sectional design, these findings should be interpreted as associations rather than causal effects, and future longitudinal or multi-source studies are warranted to strengthen inference.

### Limitations

Although this study provides useful evidence on the relationship between aging anxiety and health among middle-aged and older adults, several limitations should be noted. First, self-rated health is used as the outcome variable. While widely adopted for cross-context comparability, this measure is inherently subjective and can be biased by current affect, health expectations, or social comparisons, thus diverging from objective health status. In addition, self-rated health is ordinal. Although we reduces sparsity by merging categories and estimating regression models accordingly, this strategy may entail some information loss. Future work could combine self-rated health with objective indicators (e.g., diagnosed chronic conditions, physical examination measures, or medical records where feasible) and apply modeling approaches tailored to ordinal outcomes (e.g., ordered logit/probit or threshold-based ordinal SEM) to improve precision and comparability. Reassuringly, our robustness checks using the original five-category coding yielded results consistent with the main analyses. Second, aging anxiety is operationalized using a three-item proxy that primarily captures independence-related concerns (functional decline, loss of autonomy, and financial dependence). This operationalization does not cover other domains commonly assessed in validated multidimensional scales (e.g., appearance-related concerns, anticipated social loss, or broader existential worries), which may limit construct coverage and cross-study comparability. Future studies should validate this proxy against standard instruments and incorporate broader measures of aging anxiety when data permit. Third, the cross-sectional design precludes establishing causal directionality. Reverse causality and unobserved confounding cannot be ruled out. Longitudinal data and quasi-experimental designs are needed to strengthen causal inference and to trace the dynamic changes in aging anxiety and health over time. Fourth, the set of mediators and moderators examined in this study is not exhaustive. Due to data constraints, potentially relevant factors—such as chronic disease burden, cognitive functioning, mental health conditions, and health behaviors—could not be fully incorporated. Future research with richer measurements may further clarify additional pathways and boundary conditions. Finally, because key variables are self-reported within the same survey, common method bias remains a concern and may inflate associations due to shared response tendencies (e.g., social desirability or negative affect). Despite drawing items from different batteries and adjusting for confounders, potential measurement bias may still influence the observed effect sizes. Future research should integrate multi-source data and objective health measures, or use longitudinal designs, to further reduce this concern and strengthen inference.

## Data Availability

Publicly available datasets were analyzed in this study. This data can be found here: This study was based on a publicly available database. Dataset/questionnaire/interview used in your study has previously been published elsewhere, those informations are available through the China Survey and Data Center at Renmin University of China. The datasets generated and/or analyzed during the current study can be found at: http://cgss.ruc.edu.cn/.
