# Supplementary material for: How aging anxiety relates to self-rated health in middle-aged and older adults: the role of psychological pathways
Source: Front Psychol. 2026 Feb 19;17:1782428. doi: 10.3389/fpsyg.2026.1782428 (PMC12960128; doi:10.3389/fpsyg.2026.1782428)
Supplement: Supplementary file 1 [file Supplementary_file_1.doc]

**Appendix 1: Mediating Mechanisms and Moderating Factors of Ageing Anxiety on the Health of Middle-Aged and Older Adults**

| **Variables** | | **Weighing issues** | **Assignment** |
| --- | --- | --- | --- |
| Mediating variables | Psychological pessimism | “How often do you feel depressed or down?” | Never = 0; Rarely = 1; Sometimes = 2; Often = 3; Always= 4 |
|  | Sleep disorder | “How would you rate your sleep quality?” | Very good = 0; Relatively good = 1; Relatively poor = 2; Very poor = 3 |
|  | Loss of self-efficacy | “How confident are you in filling out medical forms such as personal profiles, medical histories, and informed consent forms?” | Very confident = 0; Confident = 1; Neither confident nor unconfident = 2; Unconfident = 3; Very unconfident = 4 |
| Moderating  variables | Social participation | This issue is measured by two questions: How often do you engage in social and recreational activities with your neighbors (such as visiting each other's homes, watching TV together, eating meals together, playing cards, etc.)? And how often do you engage in social and recreational activities with other friends (such as visiting each other's homes, watching TV together, eating meals together, playing cards, etc.)? Respondents selected one of the following options for each question: Almost daily, 1-2 times per week, Several times per month, About once per month, Several times per year, Once per year or less, Never. The study assigned values from 1 to 7 based on the frequency of each response. By calculating the average score for each response, a continuous value for this variable was derived. | The average of the sum of the frequencies of two different problems |
|  | Social support | Over the past year, has there generally been someone to listen to you talk about matters of personal concern? | No=0；Yes=1 |
|  | Insurance participation | Have you enrolled in any of the following insurance types?  Respondent's selection:  Basic Medical Insurance for Urban and Rural Residents; Basic Medical Insurance for Urban Employees; Commercial Medical Insurance  Pension Insurance; None | Participation in either insurance plan is defined as insured status, while non-participation is defined as uninsured status (Participated = 1; Not participated = 0). |
|  | Medical services utilization | How often have you visited a doctor over the past year? | Several times a week = 5  About once a week = 4  About once a month = 3  Several times a year = 2  About once a year = 1  Never = 0 |
|  | Digital technology usage | Over the past year, how have you used the following media? Respondents' choices: radio, television, internet (including mobile internet), and mobile custom messages. | Never = 1  Rarely = 2  Sometimes = 3  Often = 4  Very frequently = 5 |
|  | Social capital | The study employs social trust as a proxy variable for social capital, with the specific measurement question being: Overall, do you agree that most people in this society can be trusted? | Strongly disagree = 1  Disagree = 2  Neither agree nor disagree = 3  Agree = 4  Strongly agree = 5 |
